# Relation of Foul Air to Consumption

**Published:** 1881-08

**Authors:** P. J. Higgins


### Relation of Foul Air to Consumption.

" Experiment has shown that if an animal be kept confined in a narrow, closed apartment, so that the air supplied is always more or less vitiated by the carbonic acid which it expires, however well fed that animal may be, tubercle (consumption) will be developed in about three months." If this be the case, a large percentage of cases of consumption should be met with among the inmates of badly ventilated schools. But, fortunately, the disease is comparatively infrequent under the age of fifteen, and added to this is the protecting influence of the active exercise in the open air usually indulged in by school-children. It is upon the teachers that its blighting effects are most apparent, as they are predisposed by age, they neglect exercise in the open air, and *their mental labor is severe, and worry of mind exhausting*. Of eleven teachers who died during the last eight years within the limits of one county in Pennsylvania, two died of acute disease, one of an overdose of an habitual narcotic, and of nine attacked by consumption, eight died—six ladies and one gentleman; the other, a gentleman, will recover, at least for a time.—*From* "*Schoolroom-Ventilation,*" *by* Dr. P. J. Higgins, *in Popular Science Monthly for August.*

